# Evaluation of Candida Infection after Six Months of Transplantation in Pediatric Liver Recipients in Iran

**Published:** 2011-08-01

**Authors:** N. Honar, M. H. Imanieh, M. Haghighat, S. M. Dehghani, M. Zahmatkeshan, B. Geramizadeh, P. Badiee, S. Nikeghbalian, K. Kazemi, A. Bahador, H. Salahi, S. A. Malek-Hosseini

**Affiliations:** 1 *Gastroenterohepatology Research Center,*; 2 *Shiraz Transplant Research Center*; 3 *Clinical Microbiology Research Center, Nemazee Hospital, Shiraz University of Medical Sciences, Shiraz, Iran*

**Keywords:** Children, Liver transplantation, Late-onset Candida infection

## Abstract

Background: Liver transplantation (LT) is the standard treatment of end-stage liver diseases (ESLD). Invasive fungal infection is one of the important causes of morbidity and mortality after transplantation.

Objective: To determine the incidence of late-onset (after 6 months of LT) Candida infection in recipients.

Methods: A retrospective study was conducted to evaluate 50 pediatric patients after LT for 8 years at the LT Unit of Nemazee Hospital affiliated to Shiraz University of Medical Sciences, Shiraz, Iran. We followed the patients until 6 months post-LT for episodes of Candida infection proven by culture.

Results: One recipient (2%) developed late-onset esophageal candidiasis with improvement after intravenous amphotricin therapy but finally expired with a diagnosis of post-transplant lymphoproliferative disorder (PTLD).

Conclusions: The incidence of late-onset Candida infection is not significant in pediatric liver recipient, but it still remains a significant problem. Control of Candida colonization would reduce the risk of invasive fungal infections and possibly more fatal complications.

## INTRODUCTION

Several types of chronic liver diseases in the pediatric population need liver transplantation (LT)—the standard and life-saving treatment in this age group [1, 2]. Due to improvement in the diagnosis, surgical techniques, immunosuppressive medications and antimicrobial agents, the survival of patients have markedly improved after LT [3].

Although life-saving, LT is not curative—to get rid of a fatal disease, the recipient needs life-long consumption of immunosuppressive medications [4]. But, combination of surgical procedures and the use of immunosuppression after LT is associated with increasing risk of infection [5, 6]. One of these infections is Candida infection that can cause serious complications and affect survival. Careful monitoring and management of infections can decrease morbidity and improve the outcome of these patients [6].

Although multiple centers investigate early-onset Candia infection (during the first six months of transplantation), few studies have examined the late-onset (six months after LT) infection.

The objective of this study was to evaluate the incidence of late-onset Candida infection in pediatric liver recipients. 

## PATIENTS AND METHODS

A cross-sectional study was conducted in 50 pediatric LT patients in the LT center of Nemazee Hospital, Shiraz University of Medical Sciences (SUMS), Shiraz, Iran. 

All the studied children aged between 1 and 18 years and were followed in LT clinic, Nemazee Hospital, by LT surgeons and pediatric hepatologists for 6 to 96 months. 

If the patients had any signs or symptoms of gastrointestinal candidiasis including dysphagea, odynophagea, prolonged diarrhea without improvement, they were evaluated for Candida infections. 

Specimens were collected and cultured on Sabouraud dextrose agar (MERCK, Darmstadt, Germany) supplemented with chloramphenicol (50 mg/L) and incubated at 35 ^o^C. After 48–72 hours, if the result was positive, the colony was re-cultured for purity onto potato dextrose agar (OXOID, Ltd., Hampshire, England) and incubated at 35 ^o^C for an additional 48 hours. 

Species identification of the isolates were performed by standard methods (germ-tube formation, cornmeal for blastoconidia, pseudohyphae, and true-hyphae, as well as clamydospore production and growth on HiCrome Candida agar) and by the API 20 C system (bioMérieux, France) for sugar assimilation. 

## RESULTS

The studied patients included 32 (64%) boys and 18 (36%) girls with mean±SD age of 10.6±4.6 (range: 1–18) years. 

The most common causes of LT were extra-hepatic biliary atresia (EHBA) (n=13, 26%). Other causes of LT in our patients are presented in Table 1.

**Table 1:** Causes of liver transplantation in patients

**Diagnosis**	**n (%)**
Biliary atresia	13 (26)
Wilson's disease	8 (16)
Tyrosinemia	7 (14)
PFIC	6 (12)
Autoimmune cirrhosis	5 (10)
Cryptogenic cirrhosis	1 (2)
Criggler Najjar syndrome	1 (2)
Fulminant hepatitis	1 (2)
Congenital hepatic fibrosis	1 (2)
Primary sclerosing cholangitis	1 (2)
Neonatal hepatitis	1 (2)
Hypercholesteremia	1 (2)
Other	4 (8)
Total	50 (100)

One patient (2%) developed fever and dysphagea. She was five years old. The LT was done eight months prior to admission. She had been receiving tacrolimus for immunosuppression (with monitoring of blood level). The patient had also been receiving prednisolone that was tapered and finally discontinued. 

Due to fever and dysphagea, she was admitted to Pediatric Hepatology ward; upper endoscopy (esophago-gastro-duodenoscopy) showed multiple whitish patchy lesions suggestive of esophageal candidiasis.

Stain of esophageal brushing was showed hyphae. The specimen was also cultured (as described above), the results of which were in favor of candidiasis. Intravenous amphotricin (1 mg/kg/day for three weeks) was started for her; fever and dysphagea subsided and the patient discharged with no problem. 

The patient had a past history of acute rejection of liver; she had been admitted and responded well to pulse of methyl-prednisolone. The patient had regular follow-up visits (with monitoring of tacrolimus blood level). Finally, after about two months she again developed fever and anorexia. After admission, in physical examination, she had submandibular lymphadenopathy (LAP). Biopsy revealed post-transplantation lymphoproliferative disorder (PTLD). Unfortunately, the patient did not response to treatment and expired.

## DISCUSSION

Multiple studies showed that infection is one of the most important causes of morbidity and mortality after pediatric LT [7]. Based on these studies, the rate of infectious complications is high following LT, with a mean of 1–2.5 episodes per patient [6]. According to multiple investigations, the majority of these complications occur during the first six months after LT [8, 9]. While most infections during the first month of LT are due to pre-operative problems, those occurring 1 to 6 months after transplantation are associated with the use of immunosuppressive drugs [10].

Infection in the early post-transplant period (<1 month) is most commonly bacterial, although the risk of fungal infection is also high [8, 9]. This is the period when patients are most immunosuppressed. During this period, the most common fungal infection is with candidal species. The most common causes of infection after six months of LT are typically community-acquired pathogens, which are treated with antimicrobials commonly prescribed for non-immunosuppressed patients [5]. 

Several studies revealed that infectious complications become relatively uncommon after six months of LT (if the recipient is on a stable immunosuppressant therapy) and that these types of infections are primarily associated with chronic rejection, re-transplantation, or large doses of immunosuppressive therapy [5].

Risk factors for Candida infection after LT include complicated transplantation, repeated surgery, prolonged broad spectrum antimicrobials consumption, critically ill (ICU/dialysis), and CMV disease. These risk factors show that Candida infection usually occurs during early months after LT. Several studies showed that fungal infections commonly occur during the first six months of LT.

One study [5] revealed that Candida spp. was the only source of fungal infection and occurred in only two of 95 patients: Candida infections occurred during the first month after LT, and none of the patients developed Candida infection thereafter. 

Our study showed that one child (2%) developed late-onset Candida infection—after six months of transplantation. The incidence is somewhat higher than other studies reported. This finding may indicate that late-onset Candida infection may be an alarming sign for evaluation of more invasive complications (including PTLD). Therefore, particular attention should be paid to make the diagnosis and institute an appropriate treatment for late-onset Candida infection in pediatric patients after LT.
